# The Unicellular State as a Point Source in a Quantum Biological System

**DOI:** 10.3390/biology5020025

**Published:** 2016-05-27

**Authors:** John S. Torday, William B. Miller

**Affiliations:** 1 Department of Pediatrics, Harbor-UCLA, 1124 W. Carson Street, Torrance, CA 90502-2006, USA; 2 Independent researcher, Paradise Valley, AZ 85253, USA; Wbmiller1@cox.net

**Keywords:** point source, quantum mechanics, epigenome, unicell, niche construction

## Abstract

A point source is the central and most important point or place for any group of cohering phenomena. Evolutionary development presumes that biological processes are sequentially linked, but neither directed from, nor centralized within, any specific biologic structure or stage. However, such an epigenomic entity exists and its transforming effects can be understood through the obligatory recapitulation of all eukaryotic lifeforms through a zygotic unicellular phase. This requisite biological conjunction can now be properly assessed as the focal point of reconciliation between biology and quantum phenomena, illustrated by deconvoluting complex physiologic traits back to their unicellular origins.

## 1. Introduction

The perception of time as one-directional and asymmetric [1] is consonant with our intuitive sense of biological time, since we, as humans, witness the linear trajectory of our lives and that of those around us. However, this seemingly concrete impression may be a biological illusion. The rapidly emerging science of the Epigenome encourages a reappraisal of the significance of the previously mysterious and obligatory return of all eukaryotic organisms to the unicellular zygotic phase. In traditional terms, the zygotic unicell that results from the fertilization event of the union of two gametes following meiotic division has always been assumed to contain all of the genetic information necessary to form a new individual. As the scope of impact of epigenetics has been continually enlarged, it can now be assessed that the obligatory recapitulation of all multicellular eukaryotic organisms through the unicellular zygotic state serves a further crucial role. It is during this phase that there is critical adjustment and modulation of a stream of epiphenomena experienced within the parental macro form. It performs this action as a critical re-centering phase directed towards cellular principles and imperatives. Therefore, the unicellular zygotic phase can be considered the actual epicenter of eukaryotic life. In such circumstances, the macroorganic phase of eukaryotic life is in service to the zygotic unicell to acquire epigenetic environmental cues. The zygotic unicellular phase represents a critical transitional moment when epigenetic marks that have been acquired in the macro phase are either expressed in further development or downregulated. The importance of this regulatory mechanism becomes clearer when the actual circumstances of multicellular eukartyotic life are considered. Eukaryotic macro-organisms are multicellular units that are comprised by an intimate collaboration of innate cells and a dominant fraction of obligatory and facultative microbial life that is both mutually collaborative and competitive [2,3,4].

Under conditions in which epigenetic impacts are honored as substantial, target organisms that are acknowledged as ensembles of linked and co-dependent life experience environmental impacts as sources of ambiguity among the co-aligned constituents. It is offered that a means towards understanding epigenetic impacts in these terms is through considering the settlement of these environmental ambiguities as quantum phenomena. The centrality of this quantum action is at the level of the zygotic unicell through its unique means of arbitrating biologic space-time.

The quantum physicists Einstein, Feynman, and Bohm all thought that time was an artifact. Einstein ultimately concluded that the past, present, and future all exist simultaneously. In his book Relativity [5] Einstein stated that “Since there exists in this four dimensional structure (space-time) no longer any sections which represent ‘now’ objectively, the concepts of happening and becoming are indeed not completely suspended, but yet complicated. It appears therefore more natural to think of physical reality as a four dimensional existence, instead of, as hitherto, the evolution of a three dimensional existence. Perhaps this novel view of biology will be validated by such physics, and *visa versa*” (*sic.*).

Einstein proved that time is relative, not an absolute as classical Newtonian mechanics generally assumes. However, the majority of physicists [6] have been reluctant to relinquish any ordinary assumptions that are made about time. Physicists other than Einstein reached similar conclusions about the relative nature of time, and even made dramatic advances toward a timeless perspective of the Universe. Yet they also were unable to change the temporal mentality ingrained in mainstream physics, and society at large [7]. Richard Feynman developed the most effective and explanatory interpretation of quantum mechanics, known today as the “Sum over Histories” [8], describing time simply as a direction in space. The Sum of Histories states that the probability of an event is determined by summing together all the possible histories of that event. A particle traveling through space can move in many different ways, curved, oscillating, squiggly, and either backwards or forwards on time paths. Each of these paths has an amplitude, and when summed up in a vector, all that remain are the comparably few histories that abide by the laws and forces of Nature. In other words, Sum Over Histories indicates the direction of our ordinary clock time as simply a path in space that is more probable than any more exotic directions time might have taken otherwise.

In his book Wholeness and the Implicate Order, David Bohm [9] expresses the idea that there are two realities, the explicate and implicate. The explicate is the manner in which we conventionally perceive reality through the subjectivity of our evolved senses. Reality lies in another realm, the implicate, which he describes as a singularly continuous field. Here too, time is non-existent. Within this system other worlds are just other directions in space, some less probable, others equally as probable as the direction we experience. In a recent paper, entitled “Cosmology from the Top Down”, Stephen Hawking [10] states that “Some people make a great mystery of the multi-universe, or the Many-Worlds interpretation of quantum theory, but to me, these are just different expressions of the Feynman path integral”. The general Darwinian narrative has made no accommodation for these sources of ambiguity. However, when the zygotic unicell is appreciated as an epicenter of multicellular eukaryotic life that is fully capable of mitigating complex epigenetic inputs, then a conceptual unification of quantum phenomena with biology is its output.

## 2. Biology Meets Physics

There is a fundamental problem in reconciling the physicist’s view of time and that of the biologist. This came to light in a public debate between Einstein and Henri Bergson in Paris on 6 April 1922 [7]. Einstein advocated for the absence of time in the Universe. Bergson was adamant about the need for time in human psychology. The Einsteinian perspective was objective, based on mathematics, whereas Bergson’s biologic perspective was subjective. His perspective was the more comforting as it conformed to comfortable, entrenched, intuitive associations and correlations.

Similarly, there has been a significant shift against the prior ingrained belief that all important genetic activity is random mutational variation within a generally static central genome [11]. Since this prior viewpoint has yielded to our contemporary understanding of the scope and power of the Epigenome, a shift of similar scope between classical Newtonian physics towards a quantum world is now enabled. Such an assessment is required since the functioning genetic complement of any multicellular organism is an ever-ongoing and dynamic interrelationship between any innate species’ genome and an agitating epigenetic realm with all its diverse inputs [12,13].

Our current perception of the balance of relative importance between intrinsic participants and external impacts and their differing hereditary modes has also consequentially altered. The essential character of epigenetic influence is now acknowledged throughout evolutionary development [14]. Consequently, there is now both mandate and opportunity to critically gauge where the central control of this empowered Epigenome lies [15,16]. It can be asserted that the unicell is that centrality, uniting epigenetic influences to complex physiology and any intrinsic genome by transmuting space-time by the simultaneous knowing of both present and future.

Appreciating the primacy of the zygotic unicell in heredity depends upon two particular facets of evolutionary development that are not always considered. First, genetic assimilation into a species genome and its ultimate expression obviously differ and proceeds in a more complex manner than the seemingly direct mechanism of uncovering certain morphologic features by selective breeding [17]. Secondarily, there is now an opportunity to consider that the expression of genetic capacity is exerted through changes in both the intrinsic genome of an organism and its Epigenome as quantum phenomena [18,19]. In such circumstances, our understanding of epigenetic action is considered a “field of probabilities” that might eventually be scrupulously modeled using quantum statistical mechanics rather than a general quantum frame that lacks similar rigor [20].

Decades ago, the issue of the discontinuities of the tempo of evolution had been of particular concern to Eldredge and Gould [21]. They believed that this conflict might only be resolved by accepting that adaptation of local populations to their environment and speciation are the result of “two distinct, though interacting levels of evolution” [21]. Müller and Newman [22] have more recently considered the separability of phases in evolutionary development, “… the mechanisms of innovation and their phenotypic results—novelty—can only be properly addressed if they are distinguished from the standard evolutionary themes of variation and adaptation…”. They point out that a necessary readjustment is required, displacing an emphasis on natural selection by concentrating on the “internal dynamics of developmental systems, complementing adaptation with emergence, and contingency with inherency” [22]. From their perspective, the initiating conditions of innovation and novelty differ from their mechanistic realization and morphologic expression. The latter is represented by the balance between gene regulations of an established genome and its Epigenome. It is through this emerging contemporary understanding of the full range and power of epigenomic influences on a central genome that the necessity for an overarching regulatory apparatus becomes obvious. Absent such empowerment, genetic chaos looms.

Any epigenetic influence, no matter its type, has by definition two differing states; that is, either active or inactive in its status as a newly integrated part of an organism. Goswami makes a similar point about any genetic variations, no matter their means of induction, since initially both forms exist together in the same quantum energy state, or nearly so [23]. Clearly, given the long-standing stability of some species extending over millions of years, some means of inactivating epigenetic incursions or even intrinsic genetic mutations exists. Thus, the question then rightly becomes not only where might any regulatory agent repose within eukaryotic organisms, but how might it operate to achieve such outcomes? That agency is the zygotic unicell as the primary arbiter of the Epigenome or any modified intrinsic genetic code by adroitly collapsing the superimposition of possibilities implicit in any of multiple available states towards its environmentally appropriate explicit expression. That operative means can then be best understood as a fully quantum phenomenon.

In 2010, Goldenfield and Woese [24] proposed that treating evolution as a subset of population genetics was artificially limiting. As a result, how evolution couples with the ecological environment has not been satisfactorily explained. They asserted that the evolutionary process is best understood as a problem of non-equilibrium statistical mechanics best understood by the action of mobile genetic elements. This echoes back to an earlier critique of NeoDarwinism by Goswami. He called for reconceptualizing evolution in a quantum frame [25]. It was his contention that variation occurs at the genetic level, but natural selection acts at the morphologic level as a macro event. That relationship is never one for one.

Kauffman and Gare [26] have offered that “nature is simultaneously observing and observed and in process of becoming. But to get to this view we must surpass classical physics in which the world of actuals happen whether or not observed”. They propose that the phase space of evolution must be reconceptualized to a standpoint of always coexistent adjacent possible states that continuously constitute new boundary conditions that both impose and release evolutionary constraints. If this construct is to be construed as correct, then this new form of contingency is best understood through the concept of the collapse of the superimposition of states as that quantum mechanism. The Bohmian construct of implicates and explicates that are mutually overlapping becomes operative. Necessarily, a regulatory apparatus is, itself, implicit and it can be asserted that this is the means through which the eukaryotic zygotic unicell operates and succeeds.

It is apparent that the unicell can either express or downregulate epigentic marks [27]. This complex process must include the extent of their inclusion from either sperm or oocyte [28]. Specific factors have been identified that modulate that expression, for example 5-Methylcytosine (5mC). It has been shown that the programming of parental DNA methylomes occurs in the zygote. Those DNA methylomes, from either sperm or oocyte are significantly different [29]. An agency must reset them to compatibility in embryogenesis, and since that developmental process is obviously directly derivative of the unicellular state, it is reasonable to assume that this reprogramming is accomplished in that brief period. It is known that mammalian germ cells undergo epigenetic modifications and genomic DNA methylation, thus providing a mechanistic basis for transgenerational inheritance [16]. The programming for those transitions must emanate from the unicellular state. Although it is unlikely that the unicell is the sole locus of genomic reprogramming, actual control of it as a nexus is. These epigenetic processes are dependent upon imprinting, X chromosome inactivation, and either the permissive activity or repression of endogenous retroviruses based on nuage protein activity, piwi-interacting RNA and other post transcriptional mechanisms [30,31]. Certainly, it is known that the cells of the early mammalian embryo are, themselves, diverse and still subject to dynamic and heterogeneous epigenetic influences. Furthermore, it is clear that embryonic stem cells and primordial germ cells are epigenetically modified by transcriptional circuits that will largely determine cellular fate [32]. If any epigenetic modifiers that are present at that very early embryonic stage assert themselves, they must be derivative of the immediately juxtaposed zygotic unicellular phase as its progenitor. Therefore, as there is a firm base to the assertion that both genetic and epigenetic mechanisms govern the transition from totipotent zygote to diverse stem cell pluripotency within the embryo [15], then further too, the regulatory conditions for that interrelationship must be derivative of the prior stage.

Given the necessity for governing the Epigenome and errant mutations, recent mathematical modeling has suggested that the unicell is under the control of a quantum-like master equation that determines the informatics of environmental pressure from the Epigenome [18]. This is accomplished by adjusting the expression of epigenetic marks that always exist in the dual potential state of either expression or down-regulation and inactivation that can be appropriately be considered in the frame of the collapse of the superimposition of states. This status has been likened to a photon with its wave and particle dimensions depending on context [33], yet, always exhibiting both capacities. The history of a photon is not one of fixed chronologies but is instead its simultaneous multiple chronologies, all entangled as if all had been experienced. Therefore, in biological terms, the zygotic unicell is the sum of its chronologies that always represents more than its current physical form. Such phenomena have now superseded our conventional notion of space-time. If an epigenetic mark is conserved by the zygotic unicell, then it goes forward as both another future implicate as well as one of many present forms of a contextually dependent explicate. It may or may not be perpetually expressed dependent upon some other future situation. Therefore, as both states simultaneously exist, its past can be its future and, furthermore, contrary to any simple arrow of time, its future can settle its past in a continual state of reciprocity. It contains both future and past, in mutual coexistence such that one state collapses into expression in a manner that simultaneously reworks its past. Since both states are continuously carried as both implicate and potential explicate, any central genome is both a present and a “past” dependent on a future state, yet to be experienced. This is quantum mechanics writ biologically. The zygotic unicell can, therefore, be pictured as using eukaryotic macro organisms as settling the superimposition of states implicit to epigenetic marker activation or suppression, not only to adjust to the present, but in some ways to predict the future. This circumstance, in turn, changes its past as we, ourselves, might assess it according to our human bias. In this way, the zygotic unicell is a quantum agency, settling superimposed possibilities on a constant basis. It owes its perpetuation as a lifeform through this quantum capacity. By this means, it influences its future and its past simultaneously as a derivative of acquiring present environmental cues as epigenetic experiences via the macro form as uncollapsed possibilities and potential actualities. These experiences are then re-centered into an explicit phenotype through the recapitulated unicell. From this basis, the macro-organism re-elaborates to again experience and explore its past and future.

The observer/participant status of the eukaryotic unicell is privileged through the outward manifestation of the macro organic form that permits it to collapse the superimposition of latent states into those that are best equipped for survival. It actualizes this by taking current epigenetic marks and transforming them simultaneously into both historical and latent future heritable forms. The effect is that the current re-elaborated form is both a “present” and a forecast of future trends according to the broad parameters of homeostatic limits that are, themselves, a dynamic variable that is dependent on both current environmental status and a more enduring status within geologic parameters. In this manner, biologic organisms are successfully channeled within homeostatic constraints for the present, yet fully equipped to deal with widely shifting environments by escaping what have been traditionally supposed as biologic space-time.

In the physical world, the non-intuitive impact of quantum states to enable past and future have been experimentally shown. Physicists have studied helium atoms fired through “grates” created by lasers. The conditions of the second grate could be shown to influence how the first atom operated as it had passed through the first grate. In the quantum world it is possible for a moving object to exist in two states at once, a particle and a wave, but it is impossible to accurately measure it in both states at once. A team at the Australian National University Research School of Physics and Engineering has demonstrated that whether wave-like behavior or particle behavior is observed depends on the type of measurement at the end of the journey; that is, it is contextually determined by observer status. It is reality only when observed, so the past state can be reinterpreted by a future reality [34]. This entanglement in which future actions may influence past events has been exhibited by photon pairs [35]. In an experimental example of the flexibility of reality and time, it has been demonstrated that if two pairs of photons are entangled with each other and then separated, then the status of the photons separated from each initial pair now depends upon the status of entanglement of the two photons of the original pairs, even at a distance; the subsequent fate of the first set of separated photons can then be altered even after they have been previously measured, changed, or even destroyed [35]. In the quantum world, the future affects the past, and both are part of a predictive and probabilistic indeterminacy [36].

Quantum mechanisms offer several means of better understanding biologic processes. One of these is the actualization of Epigenomic possibilities by the collapse of the superimposition of possibilities by an entity that is both observer and participant. This is the role of the zygotic unicell exerted through the inherent properties of quantum systems as non-local correlations through entanglement at a distance and quantum coherence. Both of these latter two phenomena are fully expressed throughout the macro form. Indeed, molecular geneticist Johnjoe McFadden and quantum physicist Jim Al-Kahalili believe that all biology is quantum biology [37]. Their research indicates that the avian magnetic sense organ used to detect magnetic fields operates on a quantum basis via entanglement with molecules acting simultaneously at a distance with the final state of one molecular action determined after the fact by a subsequent one as an action without apparent connection. They further note that monarch butterflies and fruit flies use similar quantum effects in navigation, and plants are dependent on quantum processes for photosynthesis. Mae-Wan Ho has long maintained that the cell is, itself, a quantum unit and has used the human example of instantaneous muscle coordination over a scale of distances over nine orders of magnitude by the coordinated splitting and release of 10^20^ molecules of ATP [38].

When macro organisms undergo environmental and life stresses and experiences, their range of response is spread across a spectrum of potentials. This is true for newly-attached epigenetic marks. For example, Stress A might have two potential responses, type 1 and type 2. Stress B might have two differing responses, type 3 and type 4. A macro organism carries both potential responses and could possibly use any of the four types of outcomes. The zygotic unicell collapses the superimposition of possibilities in its own self-referential manner, and in so doing, is also creating its past. In effect, with respect to potential responses as latent memory, the unicell is deciding *post facto* the past of the macro form via one set of collapsed memories and not the other. In this manner, the zygotic unicell is transforming memory, creating a form of space-time shift, and in so doing is allowing individual macro-organisms to profit from the “future”, by granting a better ‘past’ through the collapse of the superimposition of differing potential states.

An analogous situation has been found in human dreams. Do we dream in color, black and white, or both? This issue has been the subject of research. People dream in both color and in black and white. Older people tend to dream more in black and white, although the majority of those dreams are still in color [39]. A study was conducted to determine whether the memory plays a role in the recollection of whether people say their dreams are black and white or in color. Research findings demonstrated that the memory of dreaming in color is related to when the dream is remembered, and the frequency of such recollections [40]. Dream recollection is subjective and context-dependent. According to any specific individual, dreams might be subjectively recorded as one or the other, or even neither. The dream, itself, is a brainwave function as a form of organic stress that is both in color and in black and white at the time it is experienced. When the individual recalls the dream, which is itself only done variously, the individual becomes an observer of its own prior experience and makes a determination that it was actually dreamed in color or in black and white. That is now a memory of a past event, solidified as one or the other. The active past had existed as a superimposition of states. The observation of that dream upon awakening, as one type or another, collapses those possibilities into memory and is then stored by the individual now stores this as past history, upon which future actions can be taken. So, in this instance, present observation is “making” the past as it shapes future actions. To the extent that the dream might influence future behavior, the past action has been in part decided on the basis of a *post facto* occurrence. This is its own form of entanglement that can be seen as roughly illustrative of the post facto determination of the actual past by a future entangled quantum event within the unicellular zygote.

The advantage of this reappraisal of evolutionary development by placing the epicentric action of the zygotic unicell within a quantum framework is that the accumulation of inputs and their activation or suppression can be identified and researched in the locus in which they actually operates. That will likely require the greater precision of the language and methods of quantum statistical mechanics. In a series of articles on the subject of evolution from unicellular to multicellular organisms, a varied integrated approach that is experimentally testable has been offered [41,42,43,44,45].

Through these deep physiologic paths, the interrelationships of space-time with environmental and epigenetic influences can be seen as a creative cellular-molecular process. Perhaps of equal importance, regarding the existence of time, the cellular-molecular approach to evolution from its origins obviates any traditional stance regarding time and space in understanding evolution.

## 3. The Origin of Complex Physiology in Space-Time

If the concept of the biological relativity of time is considered as fundamental to evolutionary development, then the origin of complex physiologic traits must be contained within that narrative. If the cellular-molecular mechanisms that have given rise to complex physiologic traits are following them from the present back to the protocell, then the temporal and spatial aspects of physiologic mechanisms become epiphenomena according to the same boundaries that have just been enumerated. For example, in the first book on this subject, entitled Evolutionary Biology, Cell-Cell Communication and Complex Disease [46], the cholesterol molecule was used as a cipher to understand evolution from its primordial origins. Cholesterol requires eleven atoms of oxygen to synthesize one molecule of cholesterol, emphasizing the requirement for atmospheric oxygen, which arose beginning in the Phanerozoic era, increasing and decreasing several times over the last five hundred million years.

The advent of cholesterol was critically important in the evolution of eukaryotes. The presence of cholesterol in the cell membrane promoted metabolism, locomotion, and respiration by allowing for a thinning of the phospholipid bilayer, giving rise to all of the enumerated traits. Subsequently, cholesterol in the cell membrane facilitated cell-cell signaling by soluble growth factors that are secreted by one cell type and to another nearby cell with a receptor embedded in such a lipid raft. By this means, receptor-mediated cell–cell signaling ultimately gave rise to a formal endocrine system.

Functional evidence for the causal relationship between cholesterol and eukaryotic evolution is seen in both the phylogenetic (long-term) and developmental (short-term) histories of cholesterol in the respiratory [46] and neuroendocrine systems [45]. Cholesterol is the most primitive of lung surfactants [47] as lipid-protein complexes that mediated the evolution of the gas exchanger from fish to amphibians, reptiles, birds, and mammals. Cholesterol is secreted by the gas gland epithelial cells lining the fish swim bladder, preventing adherence of the walls of the swim bladder to each other [48]. Cholesterol is a component of lung surfactant in subsequent forms of vertebrates associated with the water–land transition—amphibians, reptiles, mammals, and birds [47]. By tracing the structural-functional interrelationship between epithelial-mesenchymal cell-cell interactions governed by soluble growth factors and the composition and regulation of surfactant production by the alveoli, the cellular-molecular evolution of the alveolus can be followed from the swim bladder of fish to the mammalian lung [41]. That interrelationship is causal since the production of surfactant is necessary for the step-wise decrease in alveolar diameter from amphibians to reptiles, birds, and mammals [37,38,39,40,41,42]—on the one hand, the only way in which gas exchange can become more efficient is by reducing the alveolar diameter, increasing the surface area-to-volume ratio, thus enhancing gas exchange [41,42,43,44,45]. On the other hand, as evolutionary selection pressure for reduction in the alveolar diameter increases, the only way to prevent alveolar collapse due to increased surface tension based on the Law of Laplace is to produce more and/or more efficient lung surfactant. This action reduces the otherwise increasing surface tension due to the decrease in alveolar diameter, always hazarding the collapse of the alveoli [48,49,50,51]. The functional analog of surfactant facilitating oxygenation in the lung and swim bladder is cholesterol in the plasma membrane, which first facilitated oxygenation in unicellular eukaryotic organisms [45,46].

The effective culmination of lung evolution in mammals is marked by stretch-regulation of lung surfactant, providing on-demand control of this critical property of the alveolus [52]. Distension of the alveolar wall causes coordinately increased expression of both the parathyroid hormone-related protein (PTHrP) and leptin signaling mechanisms [52]. This adaptation allows for optimal regulation of surfactant production in order to avoid life-threatening atelectasis [53]. From a cellular-molecular evolutionary standpoint it offers an ontogenetic/phylogenetic link referring all the way back to unicellular organisms, as will be shown subsequently. At each point, this interchange is dependent on a critical reciprocation between environmental stresses and the macro-organism. At every stage, epigenetic implicates overlap specific explicates. There is no means by which any such complex process could have evolved without the flexibility inherent within a system based upon quantum inference of best long term environmental outcomes as buffeted by current epigenetic stresses. This is the specific regulatory role of the zygotic unicell.

In the short-term ontogenetic history of the organism, the surfactant system must mature before birth to prevent alveolar collapse [54]. Experimentally, if Scap-1, one of the genes necessary for cholesterol synthesis is deleted from the alveolar epithelial type II cell, the lung compensates by generating more lipofibroblasts in the alveolar wall, providing more surfactant to compensate for the compromised surface activity due to the loss of cholesterol synthesis by the alveolar type II (ATII) cells [55]. Thus, the lung is referring back to the exapted lipofibroblasts that it employed earlier in its phylogeny, the stored neutral lipids protecting the alveolar wall against oxidant injury [56].

In the Scap-1 deletion study, it was also found that the lipofibroblasts expressed more peroxisome proliferator activated receptor gamma (PPARγ). This refers back to the advent of the peroxisome in unicellular eukaryotes as a means of protection against oxidant stress. Under this condition, calcium stores in the endoplasmic reticulum are released under physiologic stress, putting the cell at risk of dying due to excess cytoplasmic calcium. In reaction then, the cell copes by either elaborating or adopting the peroxisome, which implements neutral lipids to buffer the excess calcium within the cell [57]. The developmental determinant of lipofibroblast differentiation is PTHrP [58], which is produced by the neighboring ATII [59]; the lipofibroblast in turn produces leptin, which acts in a retrograde fashion to stimulate surfactant synthesis by the ATIIs [55]. This cell-cell signaling mechanism is integrated by cell-surface receptors on the targeted cell-type, mediating growth factor ligand-receptor signaling by producing second messengers that induce gene expression for lung development and homeostasis [60]. Importantly then, at all stages, a critical reciprocation between the environment as a continuing set of implicates and explicates settles into biologic action. The centrality of that regulatory process is embodied in the zygotic unicell that determines those factors that will continue forward to govern embryological development.

## 4. The Evolution of Visceral Organs

The molecular mechanisms that have facilitated lung evolution are structurally-functionally homologous with other visceral organs, revealing their phylogenetic and ontogenetic origins [61]. For example, PTHrP signaling is fundamental to the skin [62], kidney [63], and skeleton [64]. In the kidney, PTHrP mediates electrolyte and fluid homeostasis within the glomerulus [65], skin barrier formation [66], and skeletal plasticity [67]. The causal nature of these interrelationships is exemplified by the deletion of PTHrP in mouse embryos, resulting in failed formation of lung alveoli [58], kidney barrier function [63], and skin barrier function [68]. The cause for this physiologic complex has come to light with the discovery that the PTHrP receptor gene duplicated during the water-land transition [69]. It has been hypothesized that the physiologic stress caused by having to adapt to land caused vascular hypertension, imparting shear stress in those particular microcirculations most affected—the lung and kidney [42,44]. Duplication of the PTHrP receptor gene would have amplified its signaling, causing increased numbers of alveoli. PTHrP promotes both epithelial [70] and endothelial cell proliferation [71]. In the kidney, the primitive glomus would have increased its PTHrP signaling, similarly resulting in expansion of the glomerular surface area to facilitate fluid and electrolyte transfer [65], as well as increasing mesangial control of urinary output [72]. In the skeleton, increased PTHrP signaling would have enhanced its bone plasticity, known as Wolf’s Law [73], allowing for the five known bone remodelings that occurred during the water-land transition, which are documented in the fossil record [74].

## 5. Mechanotransduction Points to Evolutionary Fundaments

As discussed, lung surfactant is a stretch-regulated mechanism ensuring that when the lung expands there is adequate surface activity to prevent the alveoli from collapsing on end-expiration. This process is regulated by the cell-cell signaling partners, PTHrP and leptin. The former is produced by ATIIs, the latter by lipofibroblasts. Their receptors reside on ipsilateral cells [52]. When the alveolar wall is stretched, these ligands and receptors are coordinately increased [52]. This mechanism is homologous with the swim bladder of fish [75], which also produces surfactant to lubricate the inner surface of the bladder, ensuring efficient function [48]. The swim bladder evolved in adaptation to gravity, controlling buoyancy by inflating or deflating with air to optimize swimming and feeding activities. The role of gravity was experimentally determined by putting ATIIs in free fall using a rotating wall vessel bioreactor to mimic microgravity, showing that under 0 × g conditions expression of the gene for PTHrP decreased [67]; the PTHrP receptor [52], leptin [52], and the leptin receptor [52] are also stretch-regulated, likely having evolved through positive selection for the stretch-regulated surfactant trait.

Deeper understanding of the significance of mechanotransduction derives from 0 × g exposure of yeast, the most primitive unicellular eukaryotes. Under such conditions, yeast lose their capacity to polarize and to bud [76]. Lack of polarization is a reflection of loss of calcium flux [77]. This is a fundamental metabolic property of life since budding is the mechanism of reproduction in these organisms [78]. Loss of tension on the cytoskeleton has profound effects on the physiologic status of the organism as it reflects a fundamental determination of the status of the cell as either homeostatic, mitotic or meiotic [79]. Elsewhere, for example, it has been shown that under nutrient stress, the free-swimming amoeboid form of the slime mold *Dictyostelium* generates its colonial form due to target of rapamycin (TOR) signaling to AKT [80]. TOR gene activity is regulated by nutrient availability and cytoskeletal tension [81]. Therefore, atavistic traits referring all the way back to single-celled organisms have been exapted for complex physiologic traits like alveolar homeostasis.

When such complex arcs, such as that of cholesterol or the reciprocal nature of mechanotransduction between environmental stresses and cellular proclivities, are considered the integrated nature of the mechanism of evolution can be recognized as resting upon the self-organizing and self-referential status of the unicellular state. It is this particular capacity, in combination with consistent epigenetic environmental impacts, which plays a major role in biology. Specifically, the transgenerational nature of epigenetic inheritance [82,83] can now be understood as the iterative return to the unicellular state. Therefore, rather than any superficial recounting of evolutionary processes, targets for research are readily applicable. During meiosis there is likely a form of selection for newly-acquired epigenetic marks [84]; furthermore, those marks that are inconsistent with development and homeostasis are eliminated during morphogenesis as a result of embryonic lethality [85]. However, through the intercession of the self-referential unicell and its environmental responsiveness as a quantum agency, selection is no longer random. Moreover, the compartmental characteristics of the life cycle—infancy, childhood, adolescence, adulthood, or senescence—serve to influence and act in any further expression of acquired epigenetic marks. This is an actively integrated mechanism, since epigenetic marks affect the endocrine system [86] which, in turn, determines the duration of the phases of the life cycle. It is through this reciprocating, balanced, highly integrated, iterative process that evolution prevails.

The effect of physiologic stress causing mutations and duplications was alluded to earlier. Such damage-repair processes did not occur at random; they were constrained by the structural-functional cellular niches in which radical oxygen species were generated by microvascular shear stress [87], ultimately favoring the generation of structures and functions that alleviated such stress over time [41,42,43,44,45]. At all times, the fundamental unicell remains in equipoise as a reiterative quantum agency, and by this means evolution acquires a mechanistic explanation that extends beyond random mutation and natural selection.

Such speculation is supported by the two gene duplications that occurred during the water–land transition about three hundred to five hundred million years ago, namely for the parathyroid hormone-related protein receptor (PTHrPR) [69], and the β adrenergic receptor (βAR) [88]; these duplications were accompanied by the mutation of the Mineralocorticoid Receptor to form the glucocorticoid receptor [89]. Note that all of these gene alterations targeted receptors. This is unlikely to be a coincidence. More likely, it was due to the nature of the mechanisms involved. *A priori*, it is more efficient to duplicate a receptor, which is an amplifier by nature. *A posteriori*, by virtue of the fact that the selected change was for a receptor that signals through a series of highly-evolved downstream second messengers and their target phenotypes [90], there is no disruption of the evolved homeostatic control mechanisms. Norman Horowitz had formulated a similar process [91], reasoning that if an evolving organism expended all of a given nutrient in its niche, it evolved the next enzyme in the metabolic pathway up-stream, and so on. But he did not provide a mechanism such as those above that emanate from the homeostatic constraints on the system, from its unicellular beginnings.

Such a process of receptor duplication is consistent with the observation of terminal addition, first alluded to by Ernst Haeckel in defending his Biogenetic Law [92]. Terminal addition refers to the appearance of new traits at the end of a chain of traits. However, no specific mechanism for this property has previously been speculated. In further support of this hypothesis, the other gene duplication that occurred during the water-land transition was the Goodpasture Syndrome Type IV collagen isomer [93]. It is expressed in both alveoli and glomeruli, having first appeared in amphibians and beyond during vertebrate evolution [93]. This type IV collagen isomer was advantageous because it is more hydrophobic than the other type IV collagen isotypes expressed in epithelia, preventing water loss across these barriers. However, some individuals die due to this isomer because they develop antibodies to it, inciting an immune reaction causing renal-pulmonary failure [93,94]. Perhaps anecdotally, in contradistinction to the receptor duplications, which were evolved under homeostatic control, the Goodpasture type IV collagen was not, explaining why the differences in the end results.

In any consideration of the origination of system-wide metabolic pathways or the evolutionary development of novelty, a primary issue is the identification of any impulse towards self-organization that must be present for such end-points to be observed. An appropriate answer is the feedback stigmergic loop. Stigmergy was originally described by Grasse and Parunak as a type of feedback system in which any action leaves some kind of trace in a medium [95]. Although this can pertain to abiotic systems, in biologic circumstances communicating agencies leave a trace of their action or path on the environment. This in turn generates a further action, either by the entity leaving the first trace or others that follow, which then produce other marks or different traces. In a stigmergic system, any perceivable change made in an environment by an action may trigger a subsequent action that may directly or indirectly stimulate any that follow. Heylighen [95] brought forward his own definition, “stigmergy is an indirect, mediated mechanism of coordination between actions, in which the trace of an action left on a medium stimulates the performance of a subsequent action”.

In the macro world, the best studied case is termite mounds. It is proposed that there is similar activity at the cellular level. This is an effective biologic mechanism that helps connect the causal train of physiological actions either within an organ, or by providing the necessary links between organs, *i.e.*, lung, kidney, brain, or skin. Similar gene duplications might be expressed in different environmental niches within any developing macro organism in an organized fashion. In any such instance, the individual constituents of any cellular ecology leave a stigmergic trail that empowers self-organization among individuals. Importantly, the initiating factors that result in such environmental traces can arise from a set of primary impulses that need not relate to any later developments.

## 6. The Artifactual Nature of Biologic Time

Einstein’s Theory of Relativity eliminated time as a fixed physical dimension, propelling physics from mere description to prediction. Yet within biology, time has remained a central aspect. This has hampered progress in understanding evolution. As delineated above, once morphogenesis is dialed back to the unicellular state and understood to remain rooted within that context, then the time dimension is superfluous to understanding the fundamental principles and mechanisms of physiology.

A recent breakthrough in transcending descriptive biology is the recognition of the critical importance of epigenetic inheritance in the evolutionary process and its crucial role in reproduction [83]. Indeed, mammalian reproduction is critically dependent upon several endoviral endogenizations [31,96,97,98]. Lewis Wolpert [99] has stated that “It is not birth, death or marriage, but gastrulation which is truly the most important time in your life”, gastrulation being the stage of embryogenesis during which the three embryonic germ layers are formed, dictating the further development of the fetus through interactions between these germ layers. It is now known that the genes that control the transition from the blastula to the gastrula undergo epigenetic modifications [100], offering a mechanistic basis for understanding how such epigenetic marks affect embryogenesis. Therefore, the local environment largely determines the immediate impact of epigenetic inheritance that is limited and edited during meiosis, and then again in morphogenesis as a derivative of the unicellular state. Therefore, the zygotic unicellular state must be properly appreciated as the crucial intermediary in the modulation of these epigenetic influences, not merely because that phase lies between, but as the embodiment of the reiteratively-elaborated eukaryotic organism. The obligatory return to the unicellular stage through sexual reproduction is the controlled process that permits that crucial re-centering and modulation of both the Epigenome and the intrinsic genome. It is this recapitulation through the zygotic unicell with its unique capacities that can account for both the remarkable stability over time of many species and the phenotypic plasticity of others, something that cannot be readily accommodated by a random mutation/selection model. Indeed, the proper perspective for the extent of the influence of the zygotic unicell is to look beyond the alluring structure and function of multicellular organisms and instead accept that eukaryotic organisms have never actually left that unicellular state—the myriad permutations and combinations that Francois Jacob referred to as “tinkering” [101] are merely ways in which the dominant unicell has flexibly adapted to an ever-changing environment. It is as if the unicellular state sends progeny out to the environment as agents, collecting data in the environment to inform the recapitulating unicell of ecological changes that are occurring. Through the acquisition and filtering of epigenetic marks via meiosis, fertilization, and embryogenesis, even on into adulthood, the unicell remains in effective synchrony with environmental changes.

## 7. Conclusions—The Cell as the First Niche Construction

Niche Construction Theory [102] proposes that organisms critically participate in shaping and generating their own environments. The classic example is the earthworm, which makes the soil around itself conducive to an otherwise water-adapted organism. However, the result of the excursion of any multicellular organism into the environment is the microscopic analog of niche construction, only now enacted at the cellular level. Inescapably, then, the zygotic unicell must itself be a form of niche construction within its own discrete boundaries, as is every cell. Niche construction then becomes a reiterative modular process, and by viewing evolutionary development in this manner, eukaryotic organisms can, thereby, functionally merge evolution and ecology into one continuous, harmonious process through successive layers. The crucial intercession of the zygotic unicellular phase coordinates this process by the continuous adjustment of the epigenetic impacts acquired in the macro elaboration as phenotype. The heterogeneity of observed biologic forms are, therefore, all derivative of that unicellular state and its consistent modulation. It is the continuity of the zygotic unicell and the perpetual reenactment of the eukaryotic life form through it that co-aligns biology with physics through an Einsteinian rejection of biologic time as fixed or even distinctly relevant. Biologic time must be reexamined as a quantum phenomenon lacking any rigid dimension. It, therefore, becomes largely superfluous to any consideration of eukaryotic evolutionary development. Direction does, too. Direction is always the sum of all simultaneous directional vectors that represent an always-shifting collapse of superimposed possible outcomes.

Beyond theory, there is a means for this type of process to spill forward in biologic expression without outside direction through both internally and externally generating indirect cues. In stigmergic systems, the only demand is that the participants are able to send and receive information and take action based upon it. Importantly, within such a system there is no need for planning or anticipation, memory, intentional communication, mutual awareness, simultaneous presence, imposed sequence or division of labor, or centralized control or supervision. Further, although stigmergy assumes that any participating agents are individually goal directed, it is independent of the goal itself. In biologic circumstances, it is sufficient that they seek to maintain their own homeostatic preferences. Since the individual participants will have independent goals in any mixed cellular ecology, there is a natural division of labor and the variety of these participants working together build complexity in sequence or in parallel depending upon the continuous stream of information from both within any ecological niche, or from without, based on impacting epiphenomena. Since the available information is both direct communication and non-directed chatter in which neither the sender nor the receiver is necessarily aware of the other, conflicts are diminished. The inherent power of stigmergic systems is that direct coordination among individual players is unnecessary. Through the stigmergic feedback loop, indirect information that is in one sense just the detritus of sender/receiver units coexisting in intimacy becomes useful to one or more of the players, and need not have anything to do with intentionality of the sender. In this manner, coordination emerges within tissue ecologies despite a wide assortment of constituencies through both direct and indirect means. It is particularly important to consider that unless information is expressly directed and received, it becomes a primary form of biological ambiguity. In biological terms, the resultant collapse of the superimposition of possibilities settles through the non-directed use of information, that is noise to some, and important to others, leading towards an emergent resolution of the ambiguities reflected in the totality of the information field. When resolved, some leave a trace of negative reinforcement and others become self-reinforcing. In this manner, the superimposition of possibilities is continually settled through the effective collective judgment of the ecological participants as each experiences a variety of epiphenomena within their homeostatic milieu. Importantly, as all multicellular eukaryotes are forms of coalescing mixed cellular and non-cellular participants, each constituent of the macro-organism can experience time according to its individual scope in which biologic time becomes relative. Contingency is contained within the variety of individual participants and their specialization, dependent to some degree upon the flow of internal traces and the continuous flow of external stimuli from epiphenomena. Therefore, stigmergy provides conceptual unity between the quantum ambiguities in any information system and their eventual collapse into biological expression. At every scope and scale, it is experienced as a reiterative phenomenon throughout any mixed cellular ecology and provides the effective impulse towards ecological self-organization.

Goswami maintains that any genetic variation initially exists together in the same quantum energy state, or nearly so. No collapse of those states from the Possible to the Actual need be taken. In classical Darwinism, any mutation is the immediate collapse of that superposition. In a quantum system, such a resolution does not occur until a self-referential cellular or genetic consciousness chooses among the various possibilities [25]. This self-referential capacity resides in the zygotic unicell. Through this critical intermediary, the complexity of the embryologic compartment map and eventually the mature macro-organism is embodied as a simultaneity of past, present, and future. Therefore, it is likely that biological creativity is enacted within this unicellular reprogramming phase. This transient ground state is the central locus of regulation of expression, repression, or erasure of either innate genetic and epigenetic modifications that have been experienced by the developing eukaryotic macro-organism in its own form of quantum entanglement. Anderson [103] asserts that any fundamental laws or rules that govern any simple system need not be recognizable, or necessarily apply, at other hierarchical levels. Instead, differing rules may emerge in biologic systems just as they do in abiotic states. Anderson termed that disjunction, “broken symmetry”, and it would be presumed to occur at differing scales in any complex biologic organism. As morphogenesis can now be properly construed as emanating from the single cell state and carried forward in conformity into complex multicellular life, the necessity for the regulatory oversight of the zygotic unicellular phase in eukaryotic multicellular development is underscored. Absent a consistent re-centering according to base principles in the face of accumulating epigenetic impacts, broken symmetry would mean developmental chaos. Further, then, under the broken symmetry principle, the laws that under gird simple states may not be easily recognizable in larger many body systems. Therefore, the proper unification between biology and physics will likely extend beyond any general description of quantum phenomena currently ascribed to the zygotic unicell. It can be anticipated that an exploration of its regulatory role will ultimately depend on a rigorous application of quantum statistical mechanics to the vast ensembles of hundreds of trillions of cellular and non-cellular constituents that together create any macro-organic organism.

The zygotic unicell is both nexus and point source, both observer and participant, collapsing the superimposition of some but not all Epigenomic states towards robust biologic outcomes in continuous adjustment against a background of a less acutely flexible central genome, and it is through this unique cellular agency that Bohm’s entwined realms of the explicate and implicate intimately connect, the present as past and future, welding physics and biology together in its own form of singular quantum entanglement.
